# Diagnostic Snapshot: Aggressive Malignancy or Benign Lesion?

**DOI:** 10.6004/jadpro.2014.5.4.10

**Published:** 2014-07-01

**Authors:** Paige M. Goforth

**Affiliations:** From Wellmont Cancer Institute, Kingsport, Tennessee

## History

Mrs. B. is a 67-year-old Caucasian female. She is married, works as a housewife, and has two grown children. She denies alcohol or tobacco use. Her medical history includes hypertension and poorly controlled insulin-dependent type 2 diabetes mellitus. She reports a history of frequent sunbathing from her early teens until she was in her late 40s. Her surgical history is remarkable for a hysterectomy due to advanced endometriosis. Mrs. B. does not recall whether her ovaries were removed, but she states that she has never taken hormone replacement therapy. Her dermatologic history is remarkable for a superficial solid-infiltrating basal cell carcinoma of the upper mid-back in 2000, metatypical type 2 basal cell carcinoma of the left lower medial leg in 2008, squamous cell carcinoma of the wrist in 2011, and several other precancerous lesions that were treated both topically and cryogenically.

## Chief Complaint/History of Present Illness

Mrs. B. presents today with a chief complaint of a red nodule located on her lower leg that has been present for approximately 6 months. She states that it is not painful and that it does not itch or bleed. She first noticed the nodule while vacationing at the beach. At that time, she thought it was perhaps an infected hair from shaving but says that it has not bothered her at all. She comes in today, however, because she is concerned that the nodule appears to have tripled in size seemingly "overnight." She recalls a family member with melanoma dying many years ago. Since that time, she has always been particularly concerned about getting the same type of cancer. She denies any type of trauma and is not aware of having been exposed to insects or spiders that could have bitten her. She presents with no other complaints at this time.

**Figure 1:**
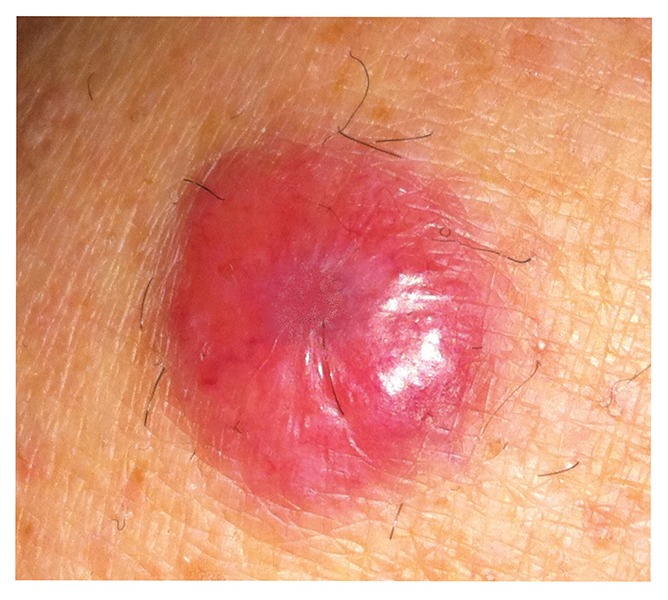
Aggressive Malignancy or Benign Lesion?

## Physical Examination

Upon physical examination, Mrs. B.’s left lower medial leg reveals a firm, 2.2-cm, violet-red, dome-shaped, solitary nodule surrounded by several tan-colored macules that are consistent with those frequently seen on the skin of patients of advanced age (see Figure 1 above). The nodule is well circumscribed and has a shiny, smooth surface. No scaling, oozing, or bleeding is visualized. No fluctuance or tenderness is noted. There are several small dry papules bilaterally, consistent with actinic keratoses. Foot exam is normal, with no ulcerations observed. The remainder of the exam is unremarkable.

## Workup

Mrs. B.’s preliminary workup included a thorough history and physical, a complete skin and lymph node examination (which revealed no palpable nodes), and a biopsy to include hematoxylin and eosin (H&E) stain as well as the appropriate immunopanel.

**Figure 2:**
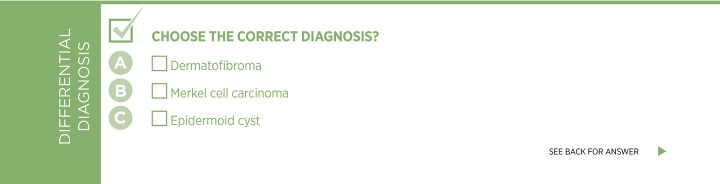
Choose the Correct Diagnosis

## Correct Answer: B 

Merkel cell carcinoma (MCC). A 3-mm punch core biopsy revealed neuroendocrine carcinoma present in the base and edges. No lymphovascular invasion was identified.

## Explanation of Incorrect Answers

**Dermatofibromas** are very common benign button-like dermal nodules that most often occur on the extremities. They are variably dome shaped but may also be depressed or "dimple" when the surrounding skin is pinched. The surface appearance may either be dull, shiny, or scaly, with borders that are not usually well defined. They can be skin-colored, pink, or brown and usually have darker more concentrated color at the center that fades toward the edge. A dermatofibroma usually presents as a solitary, firm nodule that appears gradually over several months and may not get larger at all or may spontaneously regress. Surgical removal is not necessary in these 3- to 10-mm nodules (Wolff & Johnson, 2009).

An **epidermoid cyst**, the most common cutaneous cyst, is derived from epidermis and filled with keratin and lipid-rich debris that becomes "walled off" (Wolff & Johnson, 2009). These cysts usually occur in younger patients and are commonly seen on the face, neck, upper trunk, and scrotum. The lesions usually appear as solitary nodules 0.5 to 5 cm in size. They are typically painless.

## Merkel Cell Carcinoma Basics

Merkel cell carcinoma is an aggressive neuroendocrine skin cancer that is associated with the Merkel cell polyomavirus (MCPyV) in approximately 80% to 90% of cases (Nghiem, 2013; Tilling & Moll, 2012). There is a positive correlation with survivability and a normal immune system, although 90% of patients have no known immune dysfunction at the time of diagnosis. Incidence has quadrupled over the past 20 years to an estimated 1,600 cases/year in the United States. The mortality rate exceeds that of melanoma (46%), and the 5-year survival varies from 30% to 64%. (Nghiem, 2013; Tilling, 2013).Early detection is imperative for survival.

The AEIOU mnemonic is a useful tool for remembering MCC risk factors and tumor characteristics: A = asymptomatic, E = expanding rapidly, I = immunocompromised, O = older than 50 years of age, U = ultraviolet light exposure/fair skin. Although not all patients with MCC have all five characteristics, 89% present with three or more criteria, 52% meet four or more, and 7% meet all five (Heath et al., 2008).

Merkel cell carcinoma usually appears as a solitary solid tumor. It has a high rate of recurrence following excision, spreading to the regional lymph nodes in more than 50% of patients. Disseminated disease is usually seen in the viscera and central nervous system, and less commonly in the lungs. Immunocytochemistry markers CK-7, CM2B4, LBA, Ab3, and S100 may be useful in the exclusion of other diagnostic considerations. CK-20 is a sensitive marker for MCC, whereas positive results 89% to 100% of the time, while TTF-1 is absent in MCC but positive in 83% to 100% of small cell lung cancer cases (Nghiem, 2013). This is helpful in evaluating whether a skin lesion is a MCC or a cutaneous manifestation of small cell lung cancer.

## Additional Workup

The National Comprehensive Cancer Network Clinical Practice Guidelines In Oncology (NCCN Guidelines®) recommend ruling out regional or distant metastatic disease with CT, MRI, or PET/CT of the chest, abdomen, and pelvis and also evaluating for the possibility of skin metastases from a noncutaneous primary neuroendocrine carcinoma such as small cell lung cancer, especially if CK-20 is negative. However, the most important aspect of management of clinically localized MCC continues to be evaluation of regional nodal basins with sentinel lymph node biopsy (SLNB; Brodsky, Zager, & Berman, 2011). Locoregional spread is common, so a thorough clinical exam to identify satellite lesions or palpable nodes is recommended. Consultation with a multidisciplinary tumor board experienced in treatment of MCC is also recommended.

## Management

A multidisciplinary tumor board should be consulted for improved patient outcomes. The initial gold standard of treatment is complete extirpation of the lesion preceded by SLNB if clinically indicated. For a positive SLNB, dissection of lymph nodes followed by radiation to the primary site of tumor as well as draining the lymph node basin is recommended. Adjuvant chemotherapy is not recommended as initial treatment, as it is known to lower immunity. A healthy immune system is vitally important in fighting MCC, so in most cases chemotherapy should be reserved for stage IV distant metastatic disease (M1). There are a few NCCN member institutions, however, that suggest adjuvant chemotherapy in select cases of regional disease (N1b or N2). Platinum-based therapy is the preferred regimen.

In January 2014, AMERK, a serology test, was made publicly available to patients diagnosed with MCC. Although the test is only useful in patients who are found to have anti-MCPyV T-Ag antibodies, it is 78% sensitive and 98% specific for predicting early recurrence in that population of patients (Paulson et al., 2010). More information on ordering this test may be found at www.merkelcell.org/sero. More information may be found regarding treatment guidelines, current clinical trials utilizing immunotherapy, and patient support groups at www.merkelcell.org

## Follow-Up

Close follow-up by a skilled advanced practitioner and a physician should include a complete skin and regional lymph node examination every 3 to 6 months for the first 2 years, then every 6 to 12 months after that (NCCN, 2014). Consultation with a MCC multidisciplinary team should be considered regarding individualized treatment and routine imaging. Skin self-examinations between appointments is very important.

Mrs. B. survived only 15 months after diagnosis. Recognition of the risk factors, proactive biopsy, and appropriate immunocytochemistry studies played a critical role in diagnosis and management. Mrs. B. visited the dermatologist frequently and was well versed in the ABCDEs of melanoma. Because MCC does not have the characteristic appearance of a serious malignancy, health care is often delayed. Remembering the mnemonics, advanced practitioners play a critical role in early detection by asking questions about any skin changes, conducting a thorough physical exam, performing a complete lymph node evaluation, identifying a suspicious lesion in "at-risk" patients, and proactively obtaining a biopsy.
